# Tea Consumption and Cognitive Impairment: A Cross-Sectional Study among Chinese Elderly

**DOI:** 10.1371/journal.pone.0137781

**Published:** 2015-09-11

**Authors:** Wei Shen, Yuanyuan Xiao, Xuhua Ying, Songtao Li, Yujia Zhai, Xiaopeng Shang, Fudong Li, Xiyi Wang, Fan He, Junfen Lin

**Affiliations:** 1 Department of Public Health Surveillance, Zhejiang Provincial Centre for Disease Control and Prevention, Hangzhou, Zhejiang, China; 2 Department of Chronic Disease Prevention and Control, Yuhuan Center for Disease Control and Prevention, Taizhou, Zhejiang, China; Hunter College, UNITED STATES

## Abstract

**Background:**

Laboratorial and epidemiological researches suggested that tea exhibited potential neuroprotective effect which may prevent cognitive impairment, but there were few data among the elderly aged 60 years and above in China.

**Objective:**

The objective was to explore the relationship between characteristics of tea consumption and cognitive impairment.

**Design:**

We analyzed the baseline data from Zhejiang Major Public Health Surveillance Program (ZPHS) which was conducted in 2014. Totally 9,375 residents aged 60 years and above were recruited in this study. Face-to-face interview based on a self-developed questionnaire was performed for each participant. Detailed tea consumption habits were included in the questionnaire. Cognitive impairment screening was performed by using Mini-Mental State Examination (MMSE). Education-specific cut-off points for Chinese were applied to determine the status of cognitive impairment. Logistic regression analysis was used to calculate odds ratios (ORs) of cognitive impairment associated with tea consumption.

**Results:**

The means (SD) of MMSE scores for the subjects who did not consume tea and consumed <2 cups/d, 2–4 cups/d, ≥4 cups/d were 23.3 (SD = 5.61), 23.8 (SD = 5.60), 24.5 (SD = 5.63) and 25.0 (SD = 5.08), respectively. An inverse correlation was found between tea consumption (of all types) and prevalence of cognitive impairment. Volume of tea consumption was significantly associated with cognitive impairment: compared with non-consumption participants, those who consumed < 2 cups/d, 2–4 cups/d, and ≥4 cups/d were observed ORs of 0.77 (95% CI: 0.56, 1.07), 0.62 (95% CI: 0.47, 0.81), and 0.49 (95% CI: 0.36, 0.66), respectively. Compared with non-consumption, black tea presented a positive correlation with cognitive function after controlling for potential confounders (OR = 0.52, 95% CI: 0.28, 0.95), while green tea showed no significant difference (OR = 1.04, 95% CI: 0.72, 1.51). Participants who consumed weak tea, moderate tea or strong tea more often were observed a better cognitive status when compared with those who did not have tea, with an OR of 0.51 (95% CI: 0.28, 0.92), 0.32 (95% CI: 0.19, 0.56) and 0.42 (95% CI: 0.22, 0.78) after adjusting for the potential confounders. But there was no statistically significant difference between any two of these ORs.

**Conclusion:**

Black tea consumption was association with better cognitive performance among the elderly aged 60 years and above in China, while green tea presented no correlation. The positive association of cognitive status with tea consumption was not limited to particular type of concentration.

## Introduction

With the world’s population aging, cognition-related diseases, such as mild cognitive impairment (MCI), dementia and Parkinson’s disease (PD), as an important social issue is growing. WHO announced that the number of people living with dementia worldwide is about 35.6 million and this number will be doubled by 2030, tripled by 2050 [1]. It has been indicated that many factors were positively associated with higher odds of cognitive impairment, such as low levels of education, exposure to heavy metals, with metabolic diseases, cardiovascular diseases, depression and ApoE4 gene [2–8]. Meanwhile, a lot of factors associated with lower prevalence of cognitive impairment have also been found, such as physical exercise, higher frequency of mental activity, vitamin E supplement, and tea consumption [9–14].

There were a lot of well-documented healthy beneficial components of tea, such as catechins (epicatechin-3-gallate, epigallocatechin and epigallocatechin-3-gallate, also known as EGCG), theaflavins, thearubigins, theanine and caffeine [15]. They were considered to invoke a spectrum of cellular mechanisms that can be neuroprotective [16]. Experimental and animal studies have shown that catechins (especially EGCG) can promote neural progenitor cell proliferation, improve spatial cognition learning ability and reduce *β-*amyloid mediated cognitive impairment [17–21]. Kuriyama firstly observed that in humans, a higher consumption of green tea was associated with a lower prevalence of cognitive impairment [13]. Afterwards, several studies confirmed this conclusion as well [14, 22–24].

Originating from China thousands of years ago, tea is always one of the most popular beverage for Chinese. At present, about 3 billion cups of tea are consumed per day by millions of people in the worldwide [25]. Therefore, it seems to be very critical to clarify the relationship between tea consumption and cognitive function. However, research for the elderly aged 60 years and above on the Chinese mainland is rare. Several studies have investigated the relationship between tea category and cognitive impairment or cognitive decline, but findings from these studies are inconsistent [14, 26–28]. Meanwhile, there was no paper retrieved exploring the relationship between tea concentration and cognitive function. In the present cross-sectional study, we analyzed the data of Zhejiang Major Public Health Surveillance Program (ZPHS) which is a community based cohort study focused on aging in Zhejiang Province to explore the relationship between characteristics of tea consumption and cognitive impairment.

## Materials and Methods

### Ethics statement

This study has been approved by the Ethics Committee of Zhejiang Provincial Center for Disease Control and Prevention. All participants were given free choice of receiving or rejecting the investigation. Written informed consent was obtained from each participant prior to our research.

### Study population

The data of this study was from the baseline information of Zhejiang Major Public Health Surveillance Program (ZPHS), a community based cohort study focused on aging and health among elderly in Zhejiang Province, China. Totally 7 sites were randomly selected among 90 counties/districts in Zhejiang Province, within each site, 1,500 permanent residents aged no less than 60 years were randomly selected. Totally 9,409 valid questionnaires were recovered (89.6%). After omitting several observations with incomplete information of cognitive function and tea consumption, we included 9,375 subjects in the present study.

During the baseline survey, a face-to-face interview was performed by trained professionals for each participant using a self-developed questionnaire which had been demonstrated by several epidemiologists. The information collected included demographic characteristics, family status, reproductive history, medical history, family history, behavioral risk factors, diet habits, injury, medical conditions, depression, self-care ability and cognitive function assessment. Physical examinations were also performed. The test-retest reliability ranged from 0.63 to 0.65.

### Tea consumption

Information on tea consumption during the past year was collected in the diet habits section. The main items were related to the frequency of tea consumption (the number of days consuming of tea weekly, d/w), categories of tea (included green tea, black tea, oolong tea, pu’er tea, scented tea and fruit tea), volume of tea consumption per day in the days of consuming tea (cups/d, a cup was defined as 250 ml), and preferred concentration of tea (strong, moderate and weak).

The volume of tea consumption per day (cups/d) was calculated from frequency of tea consumption and volume of tea consumption per day in the days of drinking tea. It was divided into 4 categories: non-consumption, <2 cups/d, 2–4 cups/d, and ≥4 cups/d.

### Cognitive function

Cognitive function was determined by Chinese language version of the Mini-Mental State Examination (MMSE) [29], which included 30-items. The maximum score of MMSE is 30, with higher scores indicating better cognitive function. The widely accepted cut-off score of cognitive impairment in China (Chinese Cut-off of MMSE, CCM) is education-specific: 17/18 for illiteracy, 20/21for people with 0–6 years of education, 24/25for people with more than 6 years education [30]. Commonly used MMSE cut-off worldwide was 23/24 [31]. In this study, we analyzed the data by applying both standards.

### Covariates

Other information collected included demographic characteristics (age, gender, race, education, marriage), family status (family income, having children or not), disease situation, behavioral risk factors (cigarette smoking, alcohol consumption, and physical activities,), diet habits, nutrition supplement consumption, depression, self-care ability, and physical examinations. Disease situation contained history of present illness and family history of hypertension, diabetes, hyperlipidemia, coronary heart disease (CHD), Alzheimer’s disease (AD), and PD, respectively. They were all categorized as presence or absence. Diet habits survey was to estimate the frequency of certain food intake during the past year, such as vegetables, fruits, meat, beans, and milk, etc. The frequency of food consumption was divided into 2 categories: <3 times per week; and ≥3 times per week. Depressive situation was determined by using the Patient Health Questionnaire-9 scale (PHQ-9), a 9-question version of the Primary Care Evaluation of Mental Disorders measured by self-reporting [32]. The total score was 27. It was divided into 5 categories: non-depression, 0–4; mild depression, 5–9; moderate depression, 10–14; moderately severe depression, 15–19; and severe depression, 20–27 [33].The self-care ability was determined by the Elderly Activities of Daily Living Scale, a short version of Activities of Daily Living scale (ADL). It consists of self-care tasks, such as feeding, bathing, dressing, toilet hygiene and functional mobility. The maximum score was 37. It contained 2 grades: non-dependence, 0–3; dependence, ≥4 [34]. Body mass index (BMI; as calculated from weight/ (height)^2^, kg/m^2^), waist-hip ratio (WHR; a ratio of the circumference of the waist to the hip), systolic blood pressure (SBP, mmHg) and diastolic blood pressure (DBP, mmHg) were obtained by physical examinations.

### Statistical Analysis

Characteristics of the subjects by group of different tea consumption volume were compared by using analysis of variance (ANOVA, for continuous variables), chi-square test (for dichotomous variables) or Kruskal-Wallis test (for continuous variables in skewed distribution).

Binary logistic regression was used to calculate odds ratios (ORs) of association between tea consumption and cognitive impairment, with non-consumption group treated as reference. Cognitive impairment was defined by applying two standards afore mentioned. Due to the small sample sizes of people who consumed other types of tea, we compared the difference of cognitive function between non-consumption group with black tea and green tea group. Different groups of covariates were adjusted in the regression model. Covariates in logistic regressions were chosen from the variables which were statistically associated with tea consumption in this study or clinically associated with cognitive function as revealed in previous studies (such as age, family history of AD, family history of PD, etc.).

Data analysis was conducted in SPSS 17.0. All the statistical tests we reported here were two-sided, and *P*<0.05 was accepted as statistically significant.

## Result

The age of the elders ranged from 60 to 100 years, with a mean age of 70.0 (SD = 7.69). Totally 4,827 (51.5%) respondents were female. More than a half (50.7%) were illiterate, and 92.8% elders were educated less than 6 years. The proportion of the respondents consuming tea was 27.0%, among which 77.1% drunk green tea, 19.3% drunk black tea, 1.7% drunk scented tea (Table 1).

**Table 1:** Frequency of tea consumption among study participants.

Volume	Tea category				Concentration of tea		
	Green tea (n = 1950)	Black tea (n = 487)	Scented tea (n = 43)	Other (n = 50)	Weak (n = 603)	Moderate (n = 1480)	Strong (n = 447)
<2 cups/d (%)	375(19.2)	128(26.3)	19(44.2)	24(48.0)	171(28.4)	321(21.7)	54(12.1)
2–4cups/d (%)	762(39.1)	227(46.6)	9(20.9)	13(26.0)	237(39.3)	615(41.6)	159(35.6)
≥4cups/d (%)	813(41.7)	132(27.1)	15(34.9)	13(26.0)	195(32.3)	544(36.8)	234(52.3)

Characteristics of the respondents by group of different tea consumption volume were shown in Table 2. People consuming more volume of tea were more likely to be men, minority, smoker, nonabstainer, get passive smoking, eat vegetable, red meat, eggs and beans, with better education, higher SBP, DBP,WHR, and lower BMI, were less likely to have conditions of hypertension, diabetes or CHD, and eat fruit and fish. More volume of tea consumption was associated with less frequency of physical activities, depression and cognitive impairment, but more frequency of activities of daily living and nutrition supplement. There was no statistically significant association between age, family income and tea consumption volume.

**Table 2:** Characteristics of study participants by volume of tea consumption.

Variable	Tea consumption volume				*P* ^1^
	Non-consumption (n = 6845)	<2 cups/d (n = 546)	2–4 cups/d (n = 1011)	≥4 cups/d (n = 973)	
MMSE scores, mean (SD)	23.3±5.61	23.8±5.60	24.5±5.63	25.0±5.08	<0.001
Cognitive Impairment (%)^2^	1135(16.6)	80(14.7)	141(13.9)	111(11.4)	<0.001
Cognitive Impairment (%)^3^	2703(39.5)	203(37.2)	300(29.7)	276(28.4)	<0.001
Age (y), mean (SD)	69.9±7.74	70.4±7.80	70.3±7.75	69.7±7.25	0.312
Female (%)	4204(61.4)	179(32.8)	242(23.9)	202(20.8)	<0.001
Race (%)					<0.001
Han	6716(98.1)	514(94.1)	925(93.5)	848(87.2)	
Minority	129(1.9)	32(5.9)	86(6.5)	125(12.8)	
Education (%)					<0.001
Illiteracy	3695(54.0)	248(45.4)	439(43.4)	367(37.7)	
Less than 6 years	2745(40.1)	243(44.5)	478(47.31)	484(49.7)	
More than 6 years	405(5.9)	55(10.1)	94(9.3)	122(12.6)	
Income (yuan), median	20000	25000	20000	20000	0.265
SBP (mmHg)	136.7±18.36	137.3±17.73	137.1±17.76	139.2±18.91	0.004
DBP (mmHg)	77.2±9.77	78.2±10.03	79.2±10.04	79.8±10.36	<0.001
WHR	0.90±0.068	0.91±0.070	0.91±0.062	0.91±0.071	<0.001
BMI (kg/m^2^)	23.3±3.34	23.1±3.17	22.8±3.24	22.9±3.42	<0.001
History of Present Illness					
Hypertension (%)	3155(46.1)	250(45.8)	408(40.4)	396(40.7)	<0.001
Diabetes (%)	632(9.2)	33(6.0)	59(5.8)	69(7.1)	<0.001
CHD (%)	237(3.5)	11(2.0)	24(2.4)	18(1.8)	0.007
Family History					
Hypertension (%)	1419(22.1)	104(19.5)	137(13.8)	180(18.8)	<0.001
Diabetes (%)	327(5.1)	21(4.0)	25(2.5)	34(3.5)	0.001
CHD (%)	71(1.1)	2(0.4)	2(0.2)	5(0.5)	0.009
Smoke (%)	935(13.7)	174(8.7)	421(21.0)	473(23.6)	<0.001
Passive Smoking (%)	1532(22.6)	166(30.6)	347(35.1)	406(42.4)	<0.001
Alcohol (%)	1288(18.8)	220(40.3)	387(38.3)	464(47.7)	<0.001
Exercise (%)	1399(19.6)	100(18.30)	137(13.6)	183(18.8)	<0.001
Diet (≥3 times/week) (%)					
Vegetable	6668(97.4)	525(96.2)	993(98.2)	955(98.2)	0.044
Fruit	2604(38.0)	176(32.2)	358(35.4)	268(27.5)	<0.001
Red meat	2968(43.4)	265(48.5)	579(57.3)	607(62.4)	<0.001
Fish	3220(47.0)	165(30.2)	259(25.6)	252(25.9)	<0.001
Eggs	1949(28.5)	180(33.0)	460(45.5)	387(39.8)	<0.001
Beans	3150(46.0)	225(41.2)	546(54.0)	435(44.7)	<0.001
Nutrition supplement (%)	658(9.6)	65(11.9)	104(10.3)	141(14.5)	<0.001
Depression (%)					0.001
None	6084(88.9)	495(90.7)	942(93.2)	875(89.9)	
Mild	587(8.6)	38(7.0)	53(5.2)	77(7.9)	
Moderate	133(1.9)	8(1.5)	12(1.2)	15(1.5)	
Moderately severe	26(0.4)	2(0.4)	2(0.2)	5(0.5)	
Severe	15(0.2)	3(0.5)	2(0.2)	1(0.1)	
ADL (%)					<0.001
Non-dependence	6708(98.0)	542(99.3)	1002(99.1)	965(99.2)	
Dependence	137(1.0)	4(0.5)	9(0.6)	8(0.1)	

^1^ Based on ANOVA, chi-square test or Kruskal-Wallis test.

^2^ Under the CCM of cognitive impairment.

^3^ Under the commonly used MMSE cut-off worldwide of cognitive impairment.

The result of logistic regression models for the association between tea consumption volume and cognitive impairment by applying either of two MMSE cut-offs afore mentioned are shown in Table 3. Significantly inverse association were shown between both 2–4 cups/d and ≥4 cups/d with cognitive impairment with non-consumption group treated as the reference. However, there was no statistically significant correlation between <2 cups/d group and cognitive function. After adjustment for multiple covariates, the result presented little changes. Meanwhile, the results under those two MMSE cut-offs showed little difference.

**Table 3:** Logistic regression models fitting results of the association between tea consumption volume and cognitive impairment^1^.

	Tea consumption volume				*P* ^*2*^
	Non-consumption (n = 6845)	<2 cups/d (n = 546)	2–4 cups/d (n = 1011)	≥4 cups/d (n = 973)	
Cognitive impairment, defined as CCM					
Model1^3^	1(reference)	0.86(0.68, 1.10)	0.82(0.68,0.99)	0.65(0.53,0.80)	<0.001
Model2^4^	1(reference)	0.88(0.67,1.15)	0.83(0.67,1.03)	0.69(0.55,0.87)	0.009
Model3^5^	1(reference)	0.75(0.55,1.03)	0.56(0.43,0.73)	0.42(0.31,0.56)	<0.001
Model4^6^	1(reference)	0.75(0.55,1.04)	0.58(0.44,0.77)	0.45(0.33,0.62)	<0.001
Model5^7^	1(reference)	0.77(0.56,1.07)	0.62(0.47,0.81)	0.49(0.36,0.66)	<0.001
Cognitive impairment, defined as MMSE score <24					
Model1^3^	1(reference)	0.91(0.76,1.09)	0.65(0.56,0.75)	0.61(0.52,0.70)	<0.001
Model2^4^	1(reference)	1.09(0.88,1.35)	0.71(0.59,0.84)	0.76(0.63,0.91)	<0.001
Model3^5^	1(reference)	1.09(0.86,1.39)	0.60(0.48,0.74)	0.60(0.48,0.75)	<0.001
Model4^6^	1(reference)	1.12(0.87,1.44)	0.60(0.48,0.75)	0.65(0.52,0.82)	<0.001
Model5^7^	1(reference)	1.16(0.90,1.49)	0.63(0.51,0.79)	0.68(0.54,0.86)	<0.001

^1^ Binary logistic regression analysis was used to calculate ORs and 95% CIs of the cognitive impairment related with volume of tea consumption, with non-consumption group treated as reference.

^2^P values for trend.

^3^ Crude model.

^4^ Adjusted for age, sex, race, education, marriage, tea concentration, and tea categories.

^5^ Adjusted for variables in model 2 plus physical examinations (BMI, WHR, SBP, DBP), family status (family income, have children or not) and disease situation (history of present illness and family history of hypertension, diabetes, CHD, AD, PD).

^6^ Adjusted for variables in model 3 plus behavioral risk factors (cigarette smoking, alcohol consumption, and physical activities), dietary intake (vegetables, fruits, red meat, fish, beans, milk).

^7^ Adjusted for variables in model 4 plus nutrition supplement, depression and ADL.

The association between categories of tea and cognitive impairment were shown in Table 4. The means of MMSE score were 23.3 (SD = 5.61), 24.2 (SD = 5.71), and 25.7 (SD = 4.33), respectively for the three groups below. In the unadjusted base model, it showed that black tea consumption (OR = 0.32, *P*<0.05) was significantly associated with a lower prevalence of cognitive impairment. However, there was no prominent correlation between green tea consumption (OR = 0.92, *P*>0.05) and cognitive function. After controlling for different groups of covariates, the adjusted ORs presented little changes.

**Table 4:** Logistic regression models fitting results of the association between tea categories and cognitive impairment ^1^.

	Categories of tea			*P* ^*2*^
	Non-consumption (n = 6845)	Green tea (n = 1950)	Black tea (n = 487)	
Model1^3^	1(reference)	0.92(0.80, 1.06)	0.32(0.22,047)	<0.001
Model2^4^	1(reference)	1.00(0.74,1.35)	0.48(0.29,0.80)	0.003
Model3^5^	1(reference)	0.92(0.65,1.31)	0.42(0.23,0.74)	0.004
Model4^6^	1(reference)	1.02(0.70,1.47)	0.47(0.26,0.85)	0.008
Model5^7^	1(reference)	1.04(0.72,1.51)	0.52(0.28,0.95)	0.022

^1^Binary logistic regression analysis was used to calculate ORs and 95% CIs for tea categories related to cognitive impairment which assessed with CCM, with non-consumption group treated as reference.

^2^ P value were tested by logistic regressions in which tea category was treated as categorical variable.

^3^ Crude model.

^4^ Adjusted for age, sex, race, education, marriage, tea consumption volume and tea concentration.

^5^Adjusted for variables in model 2 plus physical examinations (BMI, WHR, SBP, DBP), family status (family income, have children or not) and disease situation (history of present illness and family history of hypertension, diabetes, CHD, AD, PD).

^6^ Adjusted for variables in model 3 plus behavioral risk factors (cigarette smoking, alcohol consumption, and physical activities), dietary intake (vegetables, fruits, meat, fish, beans, milk).

^7^Adjusted for variables in model 4 plus nutrition supplement, depression and ADL.

The association between tea concentration and cognitive impairment was explored and shown in Table 5. The means of MMSE scores were 23.3 (SD = 5.61), 22.9 (SD = 6.17), 24.9 (SD = 5.22) and 25.3 (SD = 4.99) for these four groups, respectively. Compared with non-consumption group, the moderate tea (OR = 0.63, *P*<0.05) and strong tea (OR = 0.60, *P*<0.05) group were both significantly associated with a lower prevalence of cognitive impairment in the unadjusted model, while weak tea group (OR = 1.39, *P*<0.05) showed significantly correlation with a higher prevalence of cognitive impairment. In all adjusted models (model 2~model 5), the adjusted ORs of moderate tea and strong tea group compared with non-consumption group presented little changes, but weak tea group presented an inverse correlation with cognitive impairment. As well, ORs for moderate tea group were all smaller than the other two groups’. However, there was no statistically significant difference between any two of the ORs in model 5 which adjusted for all the potential confounders in this study.

**Table 5:** Logistic regression models fitting results of the association between tea concentration and cognitive impairment ^1^.

	Concentration of tea				*P* ^*2*^
	Non-consumption (n = 6845)	Weak tea (n = 579)	Moderate tea (n = 1424)	Strong tea (n = 434)	
Model1^3^	1(reference)	1.39(1.13, 1.71)	0.63(0.53, 0.76)	0.60(0.44,0.82)	<0.001
Model2^4^	1(reference)	0.48(0.29,0.80)	0.30(0.19,0.48)	0.42(0.25,0.71)	<0.001
Model3^5^	1(reference)	0.42(0.23,0.74)	0.28(0.16,0.47)	0.33(0.18,0.60)	<0.001
Model4^6^	1(reference)	0.47(0.26,0.86)	0.29(0.17,0.50)	0.38(0.21,0.70)	<0.001
Model5^7^	1(reference)	0.51(0.28,0.92)	0.32(0.19,0.56)	0.42(0.22,0.78)	<0.001

^1^Binary logistic regression analysis was used to calculate ORs and 95% CIs for cognitive impairment related with tea concentration which assessed with CCM, with non-consumption group treated as reference.

^2^ P value were determined by logistic regressions in which tea concentration was treated as non-ordinal categorical variable.

^3^Crude model.

^4^ Adjusted for age, sex, race, education, marriage, tea consumption volume and tea categories.

^5^ Adjusted for variables in model 2 plus physical examinations (BMI, WHR, SBP, DBP), family status (family income, have children or not) and disease situation (history of present illness and family history of hypertension, diabetes, CHD, AD, PD).

^6^ Adjusted for variables in model 3 plus behavioral risk factors (cigarette smoking, alcohol consumption, and physical activities), dietary intake (vegetables, fruits, red meat, fish, beans, milk).

^7^Adjusted for variables in model 4 plus nutrition supplement, depression and ADL.

## Discussion

In this large population cross-sectional study of Chinese elder aged ≥60 years, we found that tea consumption volume was associated with lower odds of cognitive impairment. It showed that black tea consumption was significantly associated with a lower prevalence of cognitive impairment. Although we did not find prominent association between green tea consumption and cognitive function. And there was no statistically significant difference between any two of the associations of cognitive function with week tea, moderate tea and strong tea. Even if almost all the ORs of cognitive impairment and moderate tea were smaller than the ORs of cognitive impairment with strong tea and weak tea.

The relationship between tea consumption volume and cognitive function was similar to most recent longitudinal or cross-sectional researches [14, 22, 26, 27]. However, by reason of all results in the present literature were based on cross-sectional study design, we cannot determine temporal relation. The inference in the present study can be made by the way that diseases related to cognitive impairment may be associated with the reduction of tea consumption behavior.

As for the association of tea categories with cognitive function, it was partly agree with an article of the Singapore Longitudinal Ageing Studies cohort in which a cross-sectional study and a cohort study was included. It found that black tea consumption was inversely associated with both cognitive impairment and cognitive decline, while green tea was only associated with less cognitive impairment [14]. In a way, we can infer that from these two studies that the positive association of black tea with less risk of cognitive function can be stronger than green tea. However, the cut-offs for MMSE used by two studies were different. But most of the existing studies indicated that green tea consumption had a positive relationship on cognitive function, and the association strength of them were stronger than the association between black tea and cognitive function. [13]. A longitudinal study of Moeko showed that green tea consumption was significantly associated with lower risk of cognitive decline, yet no relationship was found between black tea consumption and cognitive decline [26]. The short follow-up period and the small sample size might be reasons for these results. Previous studies also showed inverse association between tea consumption and cognitive impairment with no limited to particular categories of tea [27].

To our knowledge, this is the first study of human data on the association between tea concentration and cognitive impairment. We found the inverse association of cognitive impairment with tea consumption was not limited to particular type of concentration. However, we just paid our attention to the main concentration of tea consumption for the participants in the survey and ignored the situations that consumed various concentration of tea, though not many. And we collected the information about tea concentration from respondents by subjective evaluation in which information bias may exist. Thus the associations between tea concentration and cognitive impairment we found might not reflect the real relationship. In the subsequent survey, we will find some evaluation indicators which are more objective and comprehensive to assess the concentration of tea.

The neuroprotective mechanisms of tea are complex. Polyphenols maybe the most important factors which can protect the nervous system from damage by removing reactive oxygen species and reactive nitrogen, inducing the endogenous antioxidant enzymes, and binding the transition metals (such as iron and copper) [35–39]. In addition, they also regulate a variety of signal transduction pathways, including cell survival/death genes [40], and mitochondrial function which play important roles in the maintenance of neuronal viability [41]. As for black tea, the amount of catechins in black tea has been reduced due to fermentation [42, 43]. However, catechins converted into theaflavins during the fermentation process do not reduce their free radical scavenging activity [44]. Thus black tea and its components have strong antioxidant properties [45]. The strength of our study can be consolidated by the large sample size, representative sampling strategy, comprehensive questionnaire information, as well as strict quality control measures during the whole survey process.

Nonetheless, we do have certain limitations. (1) All results were based on cross-sectional study design, which means no causal-effect conclusion can be drawn. (2) Although we have adjusted for major factors that are known to be strongly relevant to cognitive function, it remains that residual confounding may still exist owing to defective measurement or incompletion of other potential confounders. (3) We collected tea consumption information from participants by self-reporting, thus the possibility of recall bias existed. And information about tea concentration was collected by subjective evaluation in which information bias may also exist. (4) We only focused on the main category of tea consumption for respondents, thus for those who consumed several categories of tea, though not many, the associations between tea consumption and cognitive impairment we reported here might not reflect the true associations.

In conclusion, habits of tea consumption, especially black tea, was associated with less prevalence of cognitive impairment. But the inverse correlation of tea consumption with cognitive impairment was not limited to particular type of concentration. In the next stage of our program, longitudinal studies will be conducted to verify these correlation and find the potential risk factors of cognitive impairment.
