# Correction: Tea Consumption and Cognitive Impairment: A Cross-Sectional Study among Chinese Elderly

**DOI:** 10.1371/journal.pone.0140739

**Published:** 2015-10-13

**Authors:** Wei Shen, Yuanyuan Xiao, Xuhua Ying, Songtao Li, Yujia Zhai, Xiaopeng Shang, Fudong Li, Xinyi Wang, Fan He, Junfen Lin

The affiliation for the second author is not indicated. Yuanyuan Xiao is affiliated with: School of Public Health, Kunming Medical University, Kunming, Yunnan, China.

The eighth author’s name is spelled incorrectly. The correct name is: Xinyi Wang.
